# Supplementary material for: Spatially Resolved Molecular Characterization of Noninvasive Follicular Thyroid Neoplasms with Papillary-like Nuclear Features (NIFTPs) Identifies a Distinct Proteomic Signature Associated with RAS-Mutant Lesions
Source: Int J Mol Sci. 2024 Dec 6;25(23):13115. doi: 10.3390/ijms252313115 (PMC11641690; doi:10.3390/ijms252313115)
Supplement: Supplementary file 1 [file ijms-25-13115-s001.zip › ijms-3304051-supplementary.pdf]

Supplementary Materials

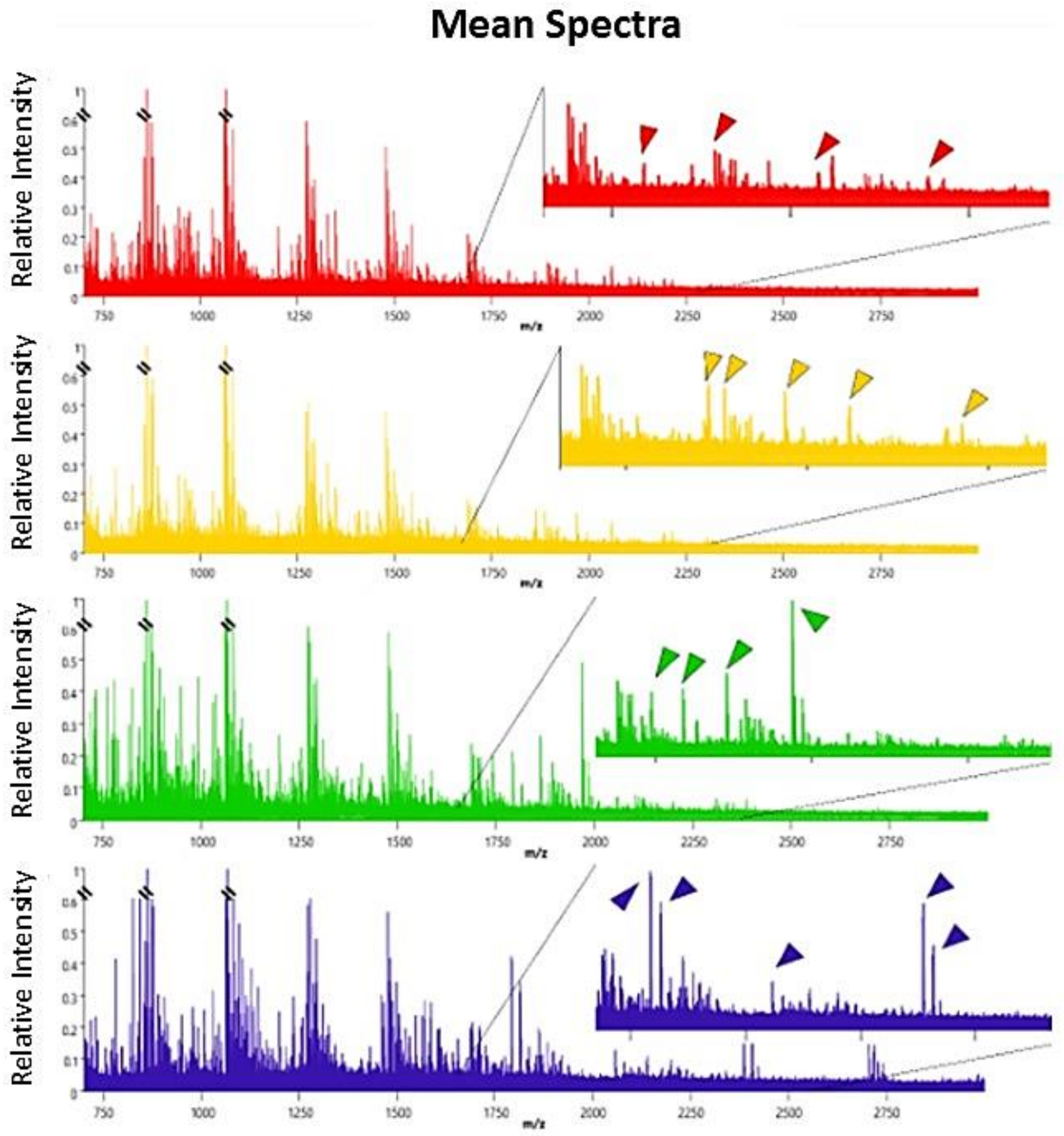

Supplementary Figure S1. Comparison of the average spectra of the segmented regions reported in Figure 1. NIFTPs (red and yellow), surrounding thyroid parenchyma (green) and fibrotic regions (purple) are shown. NIFTP, noninvasive follicular thyroid neoplasm with papillary-like nuclear features.

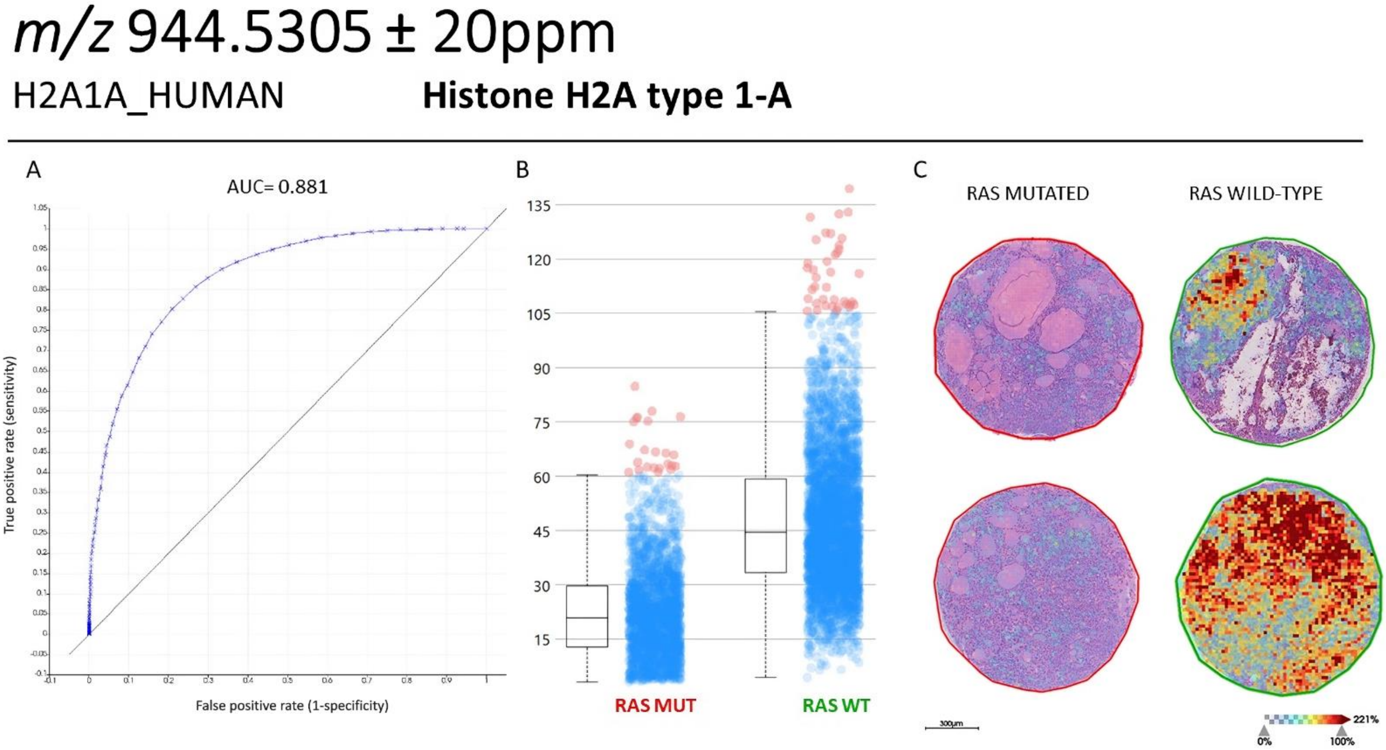

Supplementary Figure S2. Comparison of Histone H2A1A expression in RAS-mut vs. RAS-wt NIFTPs. (A-B) AUC and intensity box plots for the comparison of H2A1A expression in RAS-mut vs. 2 RAS-wt NIFTPs. (C) MALDI-MSI images showing the spatial localization of the H2A1A signal (m/z 944.5305 ± 20 ppm) in two different RAS-mut NIFTPs (left) and in two different RAS-wt NIFTPs (right). A scale bar on the bottom left is shown, as well as a color-coded scale for signal intensity. AUC, area under the curve; H2A1A, Histone H2A type 1-A; MALDI-MSI, Matrix-Assisted Laser Desorption/Ionization Mass Spectrometry Imaging; mut, mutant; NIFTP, noninvasive follicular thyroid neoplasm with papillary-like nuclear features; wt, wild-type.

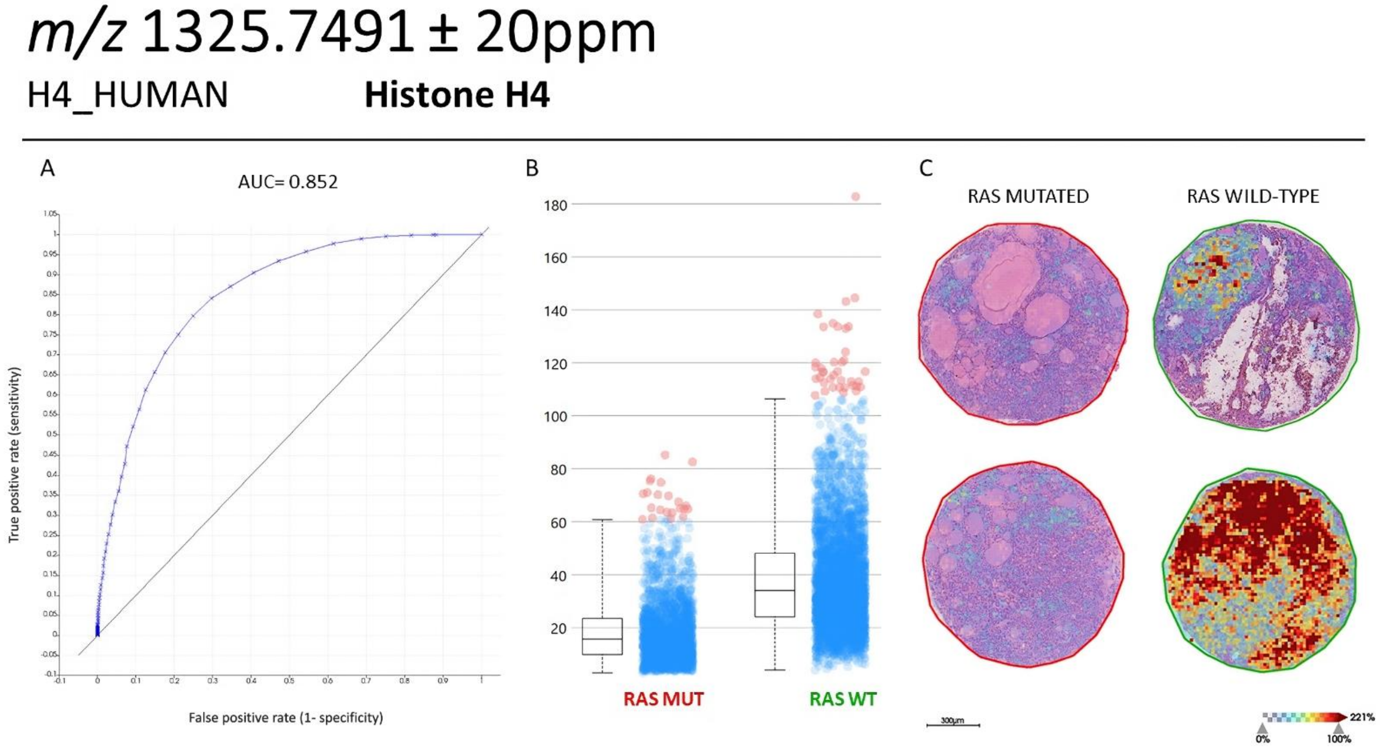

Supplementary Figure S3. Comparison of Histone H4 expression in RAS-mut vs. RAS-wt NIFTPs. (A-B) AUC and intensity box plots for the comparison of Histone H4 expression in RAS-mut vs. RASwt NIFTPs. (C) MALDI-MSI images showing the spatial localization of the H4 signal (m/z 1325.7491 ± 20 ppm) in two different RAS-mut NIFTPs (left) and in two different RAS-wt NIFTPs (right). A scale bar on the bottom left is shown, as well as a color-coded scale for signal intensity. AUC, area under the curve; MALDI-MSI, Matrix-Assisted Laser Desorption/Ionization Mass Spectrometry Imaging; mut, mutant; NIFTP, noninvasive follicular thyroid neoplasm with papillary-like nuclear features; wt, wild-type.

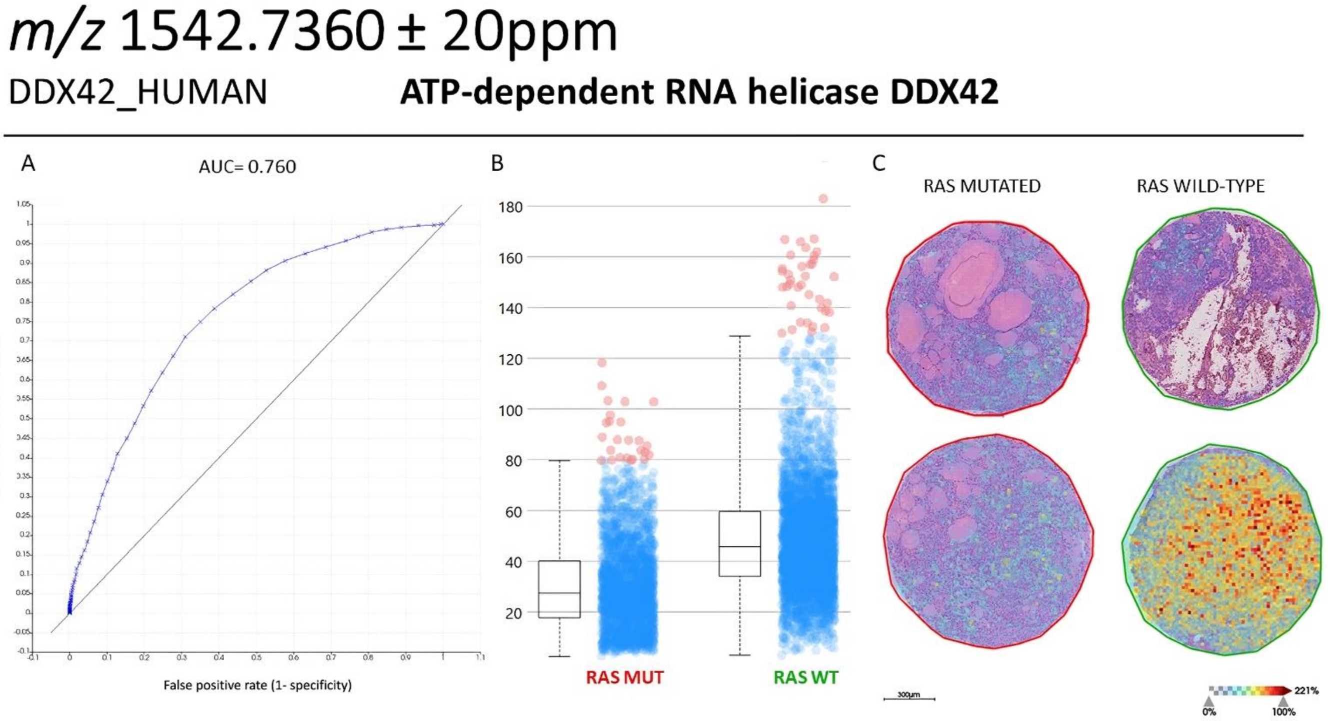

Supplementary Figure S4. Comparison of DDX42 expression in RAS-mut vs. RAS-wt NIFTPs. (A-B) AUC and intensity box plots for the comparison of DDX42 expression in RAS-mut vs. RAS-wt NIFTPs. (C) MALDI-MSI images showing the spatial localization of the DDX42 signal (m/z 1542.7360 ± 20 ppm) in two different RAS-mut NIFTPs (left) and in two different RAS-wt NIFTPs (right). A scale bar on the bottom left is shown, as well as a color-coded scale for signal intensity. AUC, area under the curve; DDX42, ATP-dependent RNA helicase DDX42; MALDI-MSI, Matrix-Assisted Laser Desorption/Ionization Mass Spectrometry Imaging; mut, mutant; NIFTP, noninvasive follicular thyroid neoplasm with papillary-like nuclear features; wt, wild-type.

Supplementary Table S1. Detailed clinicopathologic and molecular genetic features of patients enrolled in the study.

Patient No.	Age (Years)	Sex	No. of NIFTPs	Diameter (mm) *	Lobe(s) *	*RAS* mutational status	Additional mutations (where available)
1	71	M	1	5	R	WT	
2	43	F	1	25	R	WT	
3	47	F	1	7	I	WT	
4	57	F	2	19	L	MUT (*NRAS* Q61R)	
5	72	F	1	17	L	WT	
6	54	F	1	9	R	MUT (*NRAS* Q61R)	
7	56	F	1	18	R	MUT (*NRAS* Q61R)	
8	65	F	3	17	R	WT	*PTEN* p.Arg130Gln, exon 5, c.389G>A.<br>*MSH2* p.Ala189Thr, exon 3, c.565G>A.<br>*NOTCH3* p.Val644Asp, exon 12, c.1931T>A
9	58	F	1	6	L	WT	
10	46	F	1	25	L	MUT (*NRAS* Q61R)	
11	77	F	1	15	L	MUT (*NRAS* Q61R)	
12	48	M	1	12	R	MUT (*NRAS* Q61R)	

13	40	F	1	40	R	MUT (*NRAS* Q61R)	
14	55	F	1	<1	R	WT	
15	45	F	1	25	L	MUT (*NRAS* Q61R)	
16	76	F	1	15	L	WT	
17	73	M	1	4	L	WT	
18	54	M	1	2	L	WT	
19	60	M	1	23	R	WT	
20	42	F	1	35	R	WT	
21	52	F	1	10	L	WT	
22	66	M	1	17	L	WT	
23	48	F	1	10	R	WT	
24	42	M	3	9	L	MUT (*NRAS* Q61R)	
25	78	M	1	14	R	WT	
26	46	F	1	7	R	WT	
27	60	F	3	9	L	MUT (*NRAS* Q61R)	
28	44	F	1	4	L	WT	
29	70	M	3	9	R	WT	
30	32	F	1	13	R	MUT (*NRAS* Q61R)	
31	32	F	1	40	L	MUT (*NRAS* Q61R)	
32	63	F	1	5	R	WT	
33	43	F	1	18	L	WT	
34	43	F	1	38	L	WT	
35	60	F	1	9	L	WT	
36	57	F	1	17**	L	WT	
37	50	F	1	16	L	WT	
38	65	F	1	25	R	WT	
39	63	F	1	1	L	WT	
40	57	M	1	9	L	WT	
41	55	F	1	12	L	WT	
42	43	F	1	17	L	WT	
43	60	M	1	14	R	WT	
44	68	F	1	7	L	MUT (*NRAS* Q61R)	
45	58	F	1	6	R	WT	
46	69	F	1	8	L	WT	
47	58	F	2	100	L	WT	
48	55	F	1	13	R	MUT (*NRAS* Q61R)	
49	36	F	3	10	L	MUT (*NRAS* p.Gln61Arg, exon 3, c.182A>G)	*ARAF* p.Pro194Leu, exon 7, c.581C>T. *TSC2* p.Pro1589Leu, exon 37, c.4766C>T
50	55	F	1	6	L	WT	
51	21	F	1	50**	R	MUT (*KRAS* p.Gln61Arg, exon 3, c.182A>G)	*ATR* p.Met2115Ile, exon 38, c.6345G>A. *POLE* p.Leu1537Phe, exon 36, c.4609C>T
52	70	M	1	25	L	MUT (*NRAS* Q61R)	
53	49	M	5	10	R	WT	*RAD50* c.552-1G>A, exon 5, splicesite
54	51	F	1	8	R	WT	
55	65	F	1	3	L	MUT (*NRAS* Q61R)	
56	50	M	1	40	R	WT	
57	61	M	1	N.A.	R	WT	
58	56	F	1	18	L	WT	
59	34	F	1	25	R	WT	
60	35	F	1	36	R	MUT (*HRAS* p.Gln61Arg, exon 3, c.182A>G)	

61	27	F	1	55	R	MUT (*HRAS* p.Gln61Arg, exon 3, c.182A>G)	
62	80	F	1	25	L	WT	*PDGFRB* p.Glu364Lys, exon 7, c.1090G>A. *ATR* p.Met813Val, exon 11, c.2437A>G
63	43	F	1	40	R	WT	*TP53* c.560-1G>C, exon 6, splicesite. *MRE11* p.Arg488Cys, exon13, c.1462C>T. *PTEN* p.Thr277AsnfsTer21, exon 8, c.829_830insA
64	82	F	1	40	R	WT	
65	44	F	1	35	L, extending to the I	WT	*BRAF* p.Lys601Glu, exon 15, c.1801A>G. *BRCA1* p.Glu445Gln, exon 10, c.133G>C. *PALB2* p.Ala915Thr, exon 7, c.2743G>A
66	34	M	1	25	L	MUT (*HRAS* p.Gln61Arg, exon 3, c.182A>G)	*ATR* c.5739-14_5739-6delinsT, exon 34, splicesite
67	24	F	1	25	L	MUT (*NRAS* p.Gln61Lys, exon 3, c.181C>A)	
68	64	F	1	12	R	MUT (*HRAS* p.Gln61Arg, exon 3, c.182A>G)	*FANCA* p.Leu1021Trp, exon 31, c.3062T>G. *MYC* p.Asn26Ser, exon 2, c.77A>G
69	66	M	1	25	L	WT	*SLX4* p.Gly141Trp, exon 2, c.421G>T; p.Glu1159Asp, exon 12, c.3477G>C

*In cases with multiple NIFTPs, the size and location reported refer to the largest lesion

**NIFTP arisen in a cystic nodule. The reported size is the nodule diameter.

F, female; I, isthmus; L, left; M, male; MUT, mutated; N.A., not available; NIFTP, noninvasive follicular thyroid neoplasm with papillary-like nuclear features; R, right; WT, wild-type.

Supplementary Table S2. Differentially expressed MALDI-MSI *m/z* signals between *RAS*-mut and *RAS*-wt NIFTPs.

***m/z* feature**	**AUC *RAS*-wt vs. *RAS*-mut**
957.5529 m/z ± 20 ppm	0.9041812481*
971.5618 m/z ± 20 ppm	0.884147231*
944.5305 m/z ± 20 ppm	0.881027915*
943.5337 m/z ± 20 ppm	0.879509759*
1028.6182 m/z ± 20 ppm	0.856201832*
1325.7491 m/z ± 20 ppm	0.851763798*
955.5405 m/z ± 20 ppm	0.841198892*
1027.5616 m/z ± 20 ppm	0.840138047*
815.4359 m/z ± 20 ppm	0.831486259*

985.5769 m/z ± 20 ppm	0.823777903*
1070.628 m/z ± 20 ppm	0.812013322*
900.525 m/z ± 20 ppm	0.79596704*
1381.7794 m/z ± 20 ppm	0.795527666*
958.5618 m/z ± 20 ppm	0.793135711*
871.4618 m/z ± 20 ppm	0.783444604*
985.5413 m/z ± 20 ppm	0.780987255*
715.4125 m/z ± 20 ppm	0.771162483*
786.4686 m/z ± 20 ppm	0.769970473*
718.4223 m/z ± 20 ppm	0.769276108*
1542.736 m/z ± 20 ppm	0.760085363*
836.5568 m/z ± 20 ppm	0.759459543*
993.5431 m/z ± 20 ppm	0.749117775*
837.4189 m/z ± 20 ppm	0.748941547*
926.5117 m/z ± 20 ppm	0.747971099*
1363.6982 m/z ± 20 ppm	0.745725039
704.4065 m/z ± 20 ppm	0.745598223
893.4435 m/z ± 20 ppm	0.745456512
710.4202 m/z ± 20 ppm	0.740730328
712.4337 m/z ± 20 ppm	0.733864408
1092.6145 m/z ± 20 ppm	0.733608601
979.5312 m/z ± 20 ppm	0.732783994
1007.5241 m/z ± 20 ppm	0.725885786
1050.5996 m/z ± 20 ppm	0.724186564
966.511 m/z ± 20 ppm	0.722864368
1022.5377 m/z ± 20 ppm	0.722802617
1049.5451 m/z ± 20 ppm	0.722142171
894.4497 m/z ± 20 ppm	0.720295858
982.4785 m/z ± 20 ppm	0.720120391
927.5322 m/z ± 20 ppm	0.719480493
1348.7323 m/z ± 20 ppm	0.719057046
816.4553 m/z ± 20 ppm	0.718785747
907.4525 m/z ± 20 ppm	0.717211711
982.4728 m/z ± 20 ppm	0.716313016
1377.6241 m/z ± 20 ppm	0.716120698
926.5004 m/z ± 20 ppm	0.716100314
857.5039 m/z ± 20 ppm	0.716089986
1032.592 m/z ± 20 ppm	0.714531551
1187.63 m/z ± 20 ppm	0.712942621
1347.7304 m/z ± 20 ppm	0.71203338
1248.6162 m/z ± 20 ppm	0.710258764
1399.7131 m/z ± 20 ppm	0.706692084
897.5391 m/z ± 20 ppm	0.705741368
977.5222 m/z ± 20 ppm	0.705572588

793.5153 m/z ± 20 ppm	0.700953325
922.5062 m/z ± 20 ppm	0.700188512
1475.7025 m/z ± 20 ppm	0.696030801
1399.7043 m/z ± 20 ppm	0.695537179
1319.667 m/z ± 20 ppm	0.695301647
1334.6545 m/z ± 20 ppm	0.693681299
1269.6772 m/z ± 20 ppm	0.691333318
753.4341 m/z ± 20 ppm	0.687839586
1628.7879 m/z ± 20 ppm	0.686118567
770.4609 m/z ± 20 ppm	0.684986894
895.4532 m/z ± 20 ppm	0.684385318
929.5485 m/z ± 20 ppm	0.684289322
850.5719 m/z ± 20 ppm	0.683096388
1403.7335 m/z ± 20 ppm	0.682877762
1341.6513 m/z ± 20 ppm	0.682803835
1476.701 m/z ± 20 ppm	0.681924708
1491.8723 m/z ± 20 ppm	0.681902584
1013.4413 m/z ± 20 ppm	0.680442754
726.4487 m/z ± 20 ppm	0.679329563
1520.7489 m/z ± 20 ppm	0.677461996
824.4209 m/z ± 20 ppm	0.6770791
1559.7119 m/z ± 20 ppm	0.676913744
1558.7043 m/z ± 20 ppm	0.675725865
1541.8775 m/z ± 20 ppm	0.67483902
1558.6975 m/z ± 20 ppm	0.673380603
960.5454 m/z ± 20 ppm	0.670353803
1009.5104 m/z ± 20 ppm	0.669445051
988.5583 m/z ± 20 ppm	0.667651193
867.5135 m/z ± 20 ppm	0.667128381
1028.5105 m/z ± 20 ppm	0.664282647
1399.6061 m/z ± 20 ppm	0.663978679
808.4524 m/z ± 20 ppm	0.663713739
734.4131 m/z ± 20 ppm	0.663565886
850.4033 m/z ± 20 ppm	0.660848382
1385.6778 m/z ± 20 ppm	0.660155865
844.4783 m/z ± 20 ppm	0.659932075
769.4393 m/z ± 20 ppm	0.651835659
740.4039 m/z ± 20 ppm	0.651615239
1221.5503 m/z ± 20 ppm	0.651434391

**m/z* peaks with the highest discriminatory potential (AUC ≥0.75).

AUC, area under the curve; mut, mutated; NIFTP, noninvasive follicular thyroid neoplasm with papillary-like nuclear features; wt, wild-type.
